# Distributed Sensor Nodes Charged by Mobile Charger with Directional Antenna and by Energy Trading for Balancing

**DOI:** 10.3390/s17010122

**Published:** 2017-01-10

**Authors:** Celso Moraes, Sunghee Myung, Sangkeum Lee, Dongsoo Har

**Affiliations:** Cho Chun Shik Graduate School of Green Transportation, Korea Advanced Institute of Science and Technology, Daejeon 34141, Korea; asura254@kaist.ac.kr (C.M.); 1992happy@kaist.ac.kr (S.M.); sd974201@kaist.ac.kr (S.L.)

**Keywords:** hybrid charging, directional antenna, clustering, energy trading, optimal charging, mobile charger

## Abstract

Provision of energy to wireless sensor networks is crucial for their sustainable operation. Sensor nodes are typically equipped with batteries as their operating energy sources. However, when the sensor nodes are sited in almost inaccessible locations, replacing their batteries incurs high maintenance cost. Under such conditions, wireless charging of sensor nodes by a mobile charger with an antenna can be an efficient solution. When charging distributed sensor nodes, a directional antenna, rather than an omnidirectional antenna, is more energy-efficient because of smaller proportion of off-target radiation. In addition, for densely distributed sensor nodes, it can be more effective for some undercharged sensor nodes to harvest energy from neighboring overcharged sensor nodes than from the remote mobile charger, because this reduces the pathloss of charging signal due to smaller distances. In this paper, we propose a hybrid charging scheme that combines charging by a mobile charger with a directional antenna, and energy trading, e.g., transferring and harvesting, between neighboring sensor nodes. The proposed scheme is compared with other charging scheme. Simulations demonstrate that the hybrid charging scheme with a directional antenna achieves a significant reduction in the total charging time required for all sensor nodes to reach a target energy level.

## 1. Introduction

Since the inception of the Internet-of-Things, many technical challenges related to its implementation have appeared [1]. Among these challenges, provision of operating energy to the “things” (sensors) is critical from an operational point of view. A diverse range of small size, low-cost, and low-power sensors are being widely employed for a variety of applications, including mission-critical ones. Sensor nodes are often hard to access in certain mission-critical applications, such as sensors deployed to detect seismic activity [2], extreme temperatures [3], volcano activity [4], or to provide monitoring during disasters [5] and in remote environments such as the open ocean [6]. As a result, replacing sensor batteries typically involves high maintenance cost. Since loss of energy in even a single sensor node in mission-critical applications can cause serious outcomes, there is a critical need to design cost-effective methods of providing energy for sustainable sensor network operation. Among the possible solutions, wireless charging approaches, which can eliminate the costly replacement of batteries, are considered the most cost-effective [7].

The wireless charging of sensor nodes using harvested energy has been investigated in [8,9]. Fu et al. [10] proposed an optimal charging scheme using a mobile charger with an omnidirectional antenna. The charger stops at optimal charging spots to charge all sensor nodes uniformly up to a target energy level; however, time elapsed during transitions between two consecutive spots is not considered in total charging time. In addition, computational complexity increases quadratically with linearly increasing number of sensor nodes. Ding et al. [11] addressed a charging scheme for wireless sensor networks by making use of ideal magnetic resonance coils for energy transfer. Shi et al. [12] proposed a similar charging procedure using a vehicle capable of wireless power transfer through magnetic coupling to wireless sensor networks. They attempted to maximize the ratio of the designated vacation time, which is spent when the charging vehicle is at the service station, to the cycle time by using a time varying data routing algorithm. Linearization techniques were considered in this work to solve the problem. Xie et al. [13] considered a mobile charging scheme and split the service area into hexagonal cells. The charging was initiated only when the charger is located at the center of those cells. In [14], experimental charging efficiency of a radio frequency (RF) power transmitter was investigated. Lee et al. [15] inspected a dual antenna configuration for sensor nodes to enable energy harvesting and signal transmission simultaneously. Hwang et al. [16] proposed an energy harvesting scheme, in which a directional antenna was used for harvesting electromagnetic energy, and served as a reflector for a photovoltaic film. Cho et al. [17] investigated the use of a sink node with a directional antenna to prolong the lifetime of wireless sensor networks.

For some remote sensor nodes, transferring energy from overcharged nodes to undercharged nodes has been considered. Here, overcharged and undercharged sensor nodes, respectively, mean sensor nodes charged in excess and insufficiency of a target energy level. The main goal of energy trading between the sensor nodes is to achieve a balanced energy level among all the sensor nodes, to prolong the lifetime of the entire network. Xiao et al. [18] investigated an energy trading scheme based on a stochastic game theory between nodes. In their work, a sensor node is classified into either a seller or a buyer node, depending on the amount of energy it can harvest from the surrounding environment. In their paper, a seller sensor node is any overcharged sensor node, whereas a buyer sensor node is any undercharged node. The surplus energy of the seller nodes is transferred to a buyer node by many-to-one correspondence.

In the present paper, a hybrid RF charging scheme is proposed, making use of a directional antenna. It is hybrid in the sense that a major proportion of the distributed sensor nodes are overcharged by the mobile charger, using a directional antenna, and the remaining sensor nodes are then charged up to the target level through energy trading. The total charging time is the sum of the time taken for charging by the mobile charger in the first stage and the time taken for energy trading in the second stage. As it is computationally complex to consider all the distributed sensor nodes simultaneously when determining the shortest path for charging, clustering of the sensor nodes is performed by the charger prior to charging. Each cluster obtained from the clustering process consists of one cluster head and member nodes. Selection of the cluster head can be performed based on geographical consideration or energy efficiency in operation of sensor network [19]. Charging by the mobile charger is executed only for the cluster heads. This charging continues until all of the cluster heads become overcharged. Concurrently, most sensor nodes in the vicinity of the cluster heads also become overcharged. The proportion of overcharged member nodes depends significantly on the antenna gain. In the second stage, energy trading is conducted between the overcharged and the undercharged sensor nodes. Because most of the sensor nodes have already been overcharged in the first stage, only a minor proportion of the total sensor nodes must further be charged to bring them up to the target energy level.

The main contributions of the present work are summarized as follows.

1. A novel hybrid scheme for charging distributed sensor nodes by a mobile charger employing a directional antenna is proposed. For comparison of charging performance, an omnidirectional antenna is also considered. The total charging time and the total amount of energy required for all the sensor nodes to reach the target energy level are reduced compared with those of the scheme in [10]. The sequential movement of the charger with the omnidirectional antenna is determined by a golden section search (GSS), while that of the charger with the directional antenna is decided by a sector-based search. Different optimization methods are used because of the different modalities of power received by the cluster heads from the omnidirectional and directional antennas. Moreover, dependence of the total charging time on the antenna gain is discussed with simulation results.
2. A sector-based approach is employed to provide energy-efficient charging. The interior angle of the sector is determined by the beamwidth of the radiation pattern of the directional antenna. This sector-based charging reduces the charging time before energy trading. To this end, the shape of the sector is defined according to the radiation pattern.
3. The difference in time taken for energy trading, which depends on the radiation pattern of the directional antenna, is also presented. The higher directivity of the antenna is found to reduce the charging time, taken by the mobile charger, and the number of overcharged member nodes, other than cluster heads. This reduction in the number of overcharged member nodes causes an increase in the time needed for energy trading. As a result, the proportion of time taken for energy trading is larger with a higher antenna gain.

The organization of this paper is as follows. Section 2 provides the details of the hybrid charging scheme. Section 3 presents the simulation results for the proposed scheme. For comparison, the results of another charging scheme are also presented. Finally, Section 4 concludes this paper.

## 2. Hybrid Charging Scheme

### 2.1. Charging by Mobile Charger with Omnidirectional Antenna

**Figure 1:**
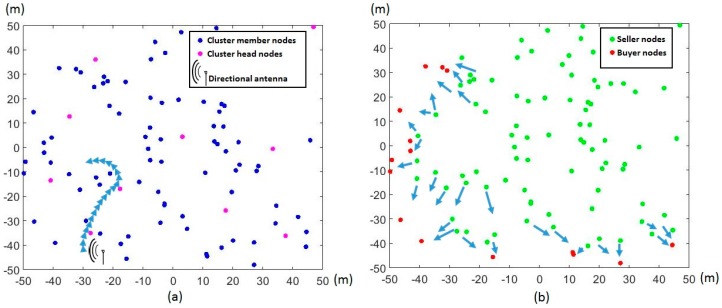
Two stages of the hybrid charging scheme: (**a**) charging by mobile charger in the first stage; and (**b**) energy trading between seller and buyer nodes in the second stage.

Figure 1a depicts the charging infrastructure sensor nodes by a mobile charger. The wireless charging model in [20] for wireless sensor networks operating at 1 GHz is given as

(1) $$p_{r}=\frac{G_{t}G_{r}\eta }{L_{p}}{\left(\frac{\lambda }{4\pi (d+\beta )}\right)}^{2}p_{0}$$

where *p_r_*, *G_t_*, *G_r_*, *η*, *λ*, *d*, *β*, *L_p_*, and *p*_0_ are the received power, antenna gain of the transmitter, antenna gain of the receiver, rectifier efficiency, wavelength, distance between the transmitter and receiver, parameter characterizing charging model, polarization loss, and transmit power, respectively. Because Equation (1) is used for the case between directional antennas, it is simplified in [10,11] by merging the parameters into a single parameter α to give an omnidirectional power equation, as follows

(2a) $$P_{r}({\vec{r}}_{j},{\vec{r}}_{charger}(t))=\frac{\alpha }{{\left(d_{j}(t)+\beta \right)}^{2}}\;j=1,...,N_{T}$$

where

(2b) $${\vec{r}}_{charger}(t)=(x_{charger}(t)\;y_{charger}(t))\;{\vec{r}}_{j}=(x_{j}\;y_{j})$$

(2c) $$d_{j}(t)=\sqrt{{\left(x_{j}-x_{charger}(t)\right)}^{2}+{\left(y_{j}-y_{charger}(t)\right)}^{2}}$$

The term $P_{r}({\vec{r}}_{j},{\vec{r}}_{charger}(t))$ in Equation (2a) represents the received power, ${\vec{r}}_{charger}(t)$ in Equation (2b) represents the two-dimensional location of the mobile charger at time *t*, and $d_{j}(t)$ is the distance between the *j*-th node located at ${\vec{r}}_{j}$ and the mobile charger.

In [10], linear programming is used to determine the optimal stopping spots of the charger with omnidirectional antenna as follows

(3) $$\min T_{cm}\;=\min\sum_{k=1}^{\infty }\Delta t_{k}$$

subject to

$$\sum_{k=1}^{\infty }\Delta t_{k}P_{r}({\vec{r}}_{j},{\vec{r}}_{charger}(t=t_{acc}))\geq ET_{S}\;j=1,...,N_{T}$$

where *T_cm_* is the time for charging by the mobile charger, $\Delta t_{k}$ is the charging duration at the *k*-th stop location, *t_acc_* is the accumulated time until the charger is located at a particular location, and $ET_{S}$ represents the target energy level of the sensor nodes. Further, *N_T_* is the total number of sensor nodes. In Equation (3), the transition of the mobile charger from one stopping spot to another is not specified. Moreover, the charging energy is evaluated for all the sensor nodes.

### 2.2. System Model of the Two-Stage Hybrid Charging Process

As in the previous studies [10,17], the infrastructure sensor nodes are considered in this work. The locations of all the sensor nodes are assumed to be known to the mobile charger. It is reasonably assumed that the sensor nodes are capable of measuring their state-of-charging (SoC). The SoC of respective sensor nodes is also known to the charger by the amount of energy transmitted to each sensor node, which can be readily calculated by the power equation (see Equation (2a)). The SoC estimation based on power equation for the mobile charger is also considered in [10] for their charging scheme.

Figure 1a depicts the situation in the first stage involving the mobile charger and the distribution of cluster heads. Figure 1b shows the initial distribution of seller and buyer nodes when the second stage begins. Unlike the mobile charger in [10], which sojourns at optimal spots without specifying the transition pattern, the mobile charger considered in this work travels while charging. The mobile charger transmits constant RF power during its travel. Furthermore, the path of sequential movements made by the mobile charger is based on the energy level of the nearby cluster heads. The first stage of the hybrid charging scheme is terminated when all the cluster heads become overcharged. Concurrently, a large number of member sensor nodes around the cluster heads also become overcharged by the mobile charger. As a result, a major proportion of the total sensor nodes has surplus energy and can sell the energy to the undercharged buyer nodes. In this second stage, the seller nodes transfer their surplus energy to the buyer nodes so that all the sensor nodes meet the target energy level. Thus, the total charging time can be expressed as

(4) $$T_{tot}=T_{cm}+T_{et}$$

where *T_tot_*, *T_cm_*, and *T_et_* represent total charging time, time for charging by the mobile charger, and time for energy trading, respectively.

Mobile charger provides energy to the rechargeable sensor network. Mobile charger stops charging as soon as all the cluster heads become overcharged. This means that the first stage is over and the second stage of energy trading is due. Note that the energy provided by the charger in the first stage is the resource of energy balancing, e.g., energy trading, in the second stage. Thus, without the mobile charger, there would be no energy to be traded between sensor nodes.

### 2.3. Clustering and Cluster Head Centric Charging in the First Stage

Popular clustering algorithms, such as the k-means algorithm [21], typically require a predetermined number of cluster heads. However, when the number of cluster heads is erroneously set, the clustering pattern deviates substantially from the visual classification.

**Figure 2:**
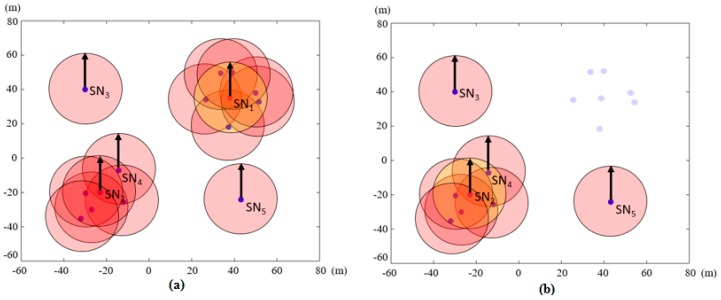
Selection of cluster heads by maximum inclusion criterion: (**a**) inclusion circles, whose centers are at sensor node locations, to obtain cluster heads; and (**b**) sensor nodes in the first cluster are not taken into account for selection of the other cluster heads.

In this paper, a heuristic clustering algorithm devised specifically for this charging method is used. The clustering of the sensor nodes is executed by the mobile charger, since it knows the locations of the sensor nodes, and does not require signaling overhead between sensor nodes and the mobile charger. Each cluster head in a cluster is chosen by a maximum inclusion criterion. An inclusion circle is placed with its center at each sensor node location. As seen in Figure 2a, the sensor nodes associated with their respective inclusion circles have an identical radius *R_cl_*. As an example of the clustering process, let us focus on seven inclusion circles around the sensor node SN_1_ in the right-top section of Figure 2a. First, the number of sensor nodes within each inclusion circle is counted and compared with the others. Within its inclusion circle the sensor node with the index SN_1_ includes seven sensor nodes (six other sensor nodes and itself), and it has the largest number of sensor nodes of all inclusion circles in the figure. For this reason, SN_1_ is chosen as the cluster head and the other six sensor nodes become member nodes of this cluster. This can be mathematically described by

(5) $${\mathrm{SN}}_{1}=\underset{i}{\arg\max}N_{R_{cl}}(i),\;i=1,...,N_{T}$$

where $N_{R_{cl}}(i)$ indicates the number of sensor nodes within the inclusion circle of the *i*-th sensor node. At this point, the cluster head SN_1_ and its associated member nodes are no longer considered for further clustering. The sensor nodes classified into the first cluster are marked by the dim-colored dots in Figure 2b. When two sensor nodes have the same number of sensor nodes within their inclusion circles, the first cluster head is selected randomly. This procedure is then repeated to select the second cluster head, whose inclusion circle includes the largest number of remaining sensor nodes. Whole process of finding cluster heads is completed when all the cluster heads, the *N_cl_* cluster heads, are found. Some sensor nodes such as SN_3_ and SN_5_ in the figure form single node clusters. As the cluster heads are mostly located at the centers of geographically grouped sensor nodes, the cluster head centric charging affects most of the member nodes, for both omnidirectional and directional transmissions of energy. In [22], a similar heuristic clustering method was used. For clustering, a grid is formed and only some grid points are chosen as the stopping positions of the mobile sink to collect the data from sensors. These stopping points can be considered locations of virtual cluster heads. Thus, stopping positions are not sensor node locations. In the case of our clustering method, locations of cluster heads are locations of some sensor nodes. In addition, in [22], a post processing to eliminate the redundant stopping positions was executed whereas our method has no such post processing.

### 2.4. Charging by Mobile Charger with Directional Antenna

The power Equation (2a) can be considered to be effective between omnidirectional antennas, corresponding to 0 dB antenna gain here. Equation (2a) can be modified to consider the directional antenna of the mobile charger. The directional antenna has a directive gain, which is the ability to concentrate the transmitted energy in a particular direction or orientation [23,24]. The use of directional antennas has been proven efficient for communications in fading situations. In [25], a directional antenna was considered in a WiMAX communication system attached to the rooftop of a moving car, and demonstrated the advantage of directional antenna in relation to omnidirectional antenna for outage and bit error rate (BER) in a Nakagami-m fading channel. In [26] a directional antenna was utilized for a relay scenario with a Rayleigh fading channel, and it was shown that the outage probability can be improved with the variation of the gain and beamwidth.

Let *φ* = 0° be the direction corresponding to the antenna gain and let *φ_j_*(*t*) be the orientation angle *φ* of the *j*-th sensor node at time *t*. Owing to the mobility of the charger, the orientation angle of the *j*-th infrastructure sensor node is a function of time *t*. Then, the power equation with directive gain $g_{A}(\phi_{j}(t))$ in the linear scale, where the antenna type *A* denotes the specific antenna gain, is given by

(6) $$P_{r}^{A}({\vec{r}}_{j},{\vec{r}}_{charger}(t),\phi_{j}(t))=g_{A}(\phi_{j}(t))\frac{\alpha }{{\left(d_{j}(t)+\beta \right)}^{2}}$$

**Figure 3:**
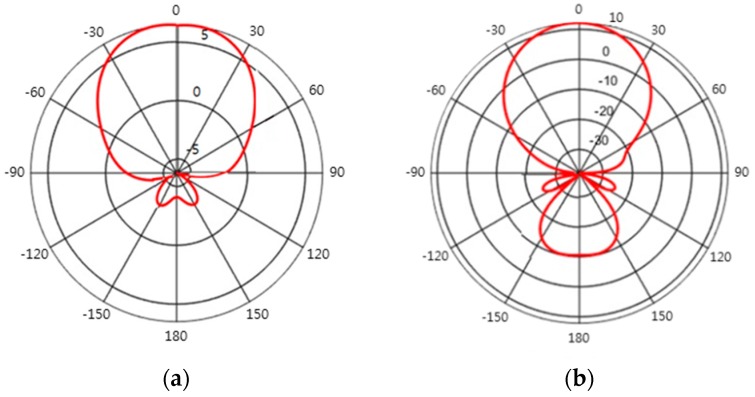
Directive gain g*_A_*(*φ*) of different types of directional antennas according to the orientation angle *φ*: (**a**) antenna gain = 6 dB; and (**b**) antenna gain = 12 dB.

Figure 3 presents the radiation patterns of the different types of directional antennas used in this work. The 6 dB antenna gain is obtained by a helical antenna and the 12 dB antenna gain is obtained with a patch array antenna. Note that the directive gain in dB in Figure 3 is relative to the implicit transmit antenna gain in (2a). Helical antennas are known for their high directivity and circular polarization [27], and patch array antennas have many advantages including low cost, light weight, and low profile [28]. However, as our main concern is to investigate the impact of directive gain on charging performance, the details of antenna design are beyond the scope of this paper. 

The directive gain $g_{A}(\phi_{j}(t))$ in (6) is appropriate for a far-field zone. The typical criterion for separating far and near-field zones is $d_{j}(t)>2D^{2}/\lambda$ [28], where *D* is the maximum dimension of the antenna or array antenna and λ is the wavelength. Considering typical RF frequencies of 1~2 GHz [22] for wireless rechargeable sensor nodes and the small *D* (~0.5 m) of helical and patch array antennas designed in this work for such transmit frequencies, the far-field zone starts from several meters of $d_{j}(t)$. Because the works in [10,11] did not consider zone separation with power Equation (2a), power Equation (6) is also assumed effective regardless of $d_{j}(t)$ values. The half-power (3 dB attenuation) beamwidths (≡2B) of antenna gains 6 dB and 12 dB are 100° and 44°, respectively. These radiation patterns are in the typical shapes of the respective antenna gains. However, the lack of analytic expressions in the radiation patterns in Figure 3 considerably complicates the derivation of the optimal charging path. Therefore, a suboptimal and sequential approach to charge the cluster heads is attempted in this paper.

### 2.5. Sequential Sector-Based Charging by Mobile Charger

The path taken by the mobile charger is determined by the distribution of cluster heads. The charging scheme in [10] considers all the sensor nodes in the network to find the optimal charging spots, but this approach introduces high computational complexity which grows quadratically with the number of sensor nodes.

Let the objective function $T_{cm}^{A}(PATH_{i})$ be the charging time *T_cm_* taken along a path *PATH_i_*, parameterized by an antenna type *A*. Then, the goal of optimization is to minimize the $T_{cm}^{A}(PATH_{i})$ as follows

(7a) $$T_{cm,\min}^{A}=\underset{i}{\min}T_{cm}^{A}(PATH_{i})\;\forall paths$$

subject to

(7b) $$\sum_{n=1}^{N_{PATH_{i}}}\int_{(n-1)\Delta t}^{n\Delta t}P_{r}^{A}({\vec{r}}_{j},\;{\vec{r}}_{charger}(t),\;\phi_{j}^{\prime }(t))dt\geq EL_{\min}^{A}\;j=1,...,N_{cl}$$

where the *N_cl_* is the number of cluster heads and $P_{r}^{A}({\vec{r}}_{j},\;{\vec{r}}_{charger}(t),\;\phi_{j}^{\prime }(t))$ represents the power received by the *j*-th cluster head located at ${\vec{r}}_{j}$ transmitted from the charger at time *t* and $\phi_{j}^{\prime }(t)$ is the orientation angle of the *j*-th cluster head with respect to the direction of charger movement. Equation (7b) shows that the travelling of the mobile charger is modeled by the concatenated discrete movements and each movement, staying at the same location or shifting to a new location, is made in every time interval ∆*t*. The parameter $EL_{\min}^{A}$ represents the target energy level of the cluster heads for antenna type *A*. As the number of seller nodes during energy trading depends on antenna type *A*, the $EL_{\min}^{A}$ is adjusted to ensure that the cluster heads have enough energy to transmit to the buyer nodes. The $EL_{\min}^{A}$ set for the cluster heads is different from the target energy level *ET_s_* set for all the sensor nodes. The $EL_{\min}^{A}$ is set higher than the *ET_s_* so that all the sensor nodes meet *ET_s_* at the end of energy trading. Note that the charging time $T_{cm}^{A}(PATH_{i})$ depends on the starting point of the path of the mobile charger. The $N_{PATH_{i}}$ indicates the number of time intervals ∆*t* associated with the path *PATH_i_* and the $N_{PATH_{i}}$ includes the number of time intervals during which the charger stays at the same location. When comparing Equations (3) and (7a), it is noted that the transition of the mobile charger is not specified by [10], whereas our approach is based on sequential movements of the charger. In addition, the charging energy is evaluated for all the sensor nodes in [10], whereas in this work it is evaluated only for the cluster heads.

**Figure 4:**
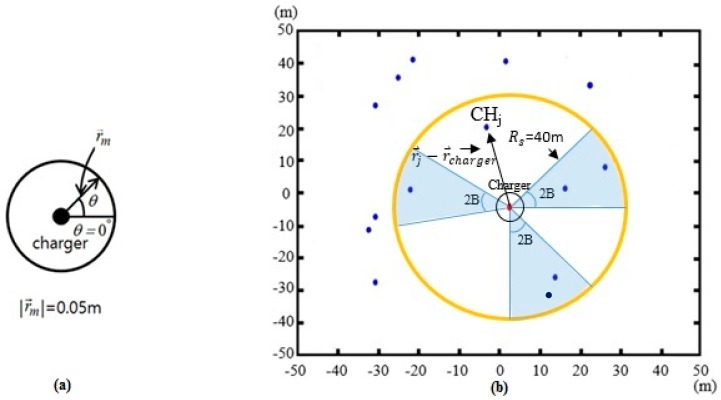
Next location circle and service sector for charging undercharged cluster heads: (**a**) next location circle; and (**b**) sectors including undercharged cluster heads.

For each time interval ∆*t*, a discrete action is performed by the charger. The possible discrete actions of the mobile charger at a location are either “staying at the same location” or “shifting to the next optimal location”. Figure 4a shows the “next location circle” on which the next optimal location of the charger is placed. The charger can stay at the same location as the next discrete movement as long as the location is optimal. The time interval ∆*t* for making a decision on the next movement is set to 2.5 ms and the distance *r_m_* between the current location and next optimal location is set to 0.05 m. If the next discrete movement to the next location circle occurs over a sizable time, and the location of the mobile charger is continuously shifted accordingly, the velocity of the mobile charger is (0.05 m/2.5 ms =) 72 km/h. While the next location circle is concerned with the location of the mobile charger, the service sector with the radius *R_s_* shown in Figure 4b is used to evaluate the amount of power received by the undercharged cluster heads, thus influencing the decision regarding the next optimal location on the next location circle. Undercharged cluster heads are considered, when making a decision on the next action, to overcharge them as quickly as possible to prepare for energy trading. The directive gain of the directional antenna can be leveraged to limit the number of undercharged cluster heads being considered in the decision of the charger movement direction. The interior angle of each service sector is 2B, and the bisector of the interior angle connects the charger and an undercharged cluster head. Therefore, sector size is increased as the antenna gain is decreased. In the case of an omnidirectional antenna, 2B is set to 360°.

Let $\theta_{i}(t),$ *i* = 1,...,$N_{uch}(t),$ where $N_{uch}(t)$ is the total number of undercharged cluster heads at time *t*, be the θ of the *i*-th undercharged cluster head at time *t*. The $\theta_{i}(t)$ is formed by the horizontal line and the line connecting the charger and the *i*-th undercharged cluster head, as shown in Figure 4a. Each $\theta_{i}(t)$ is associated with a sector. For an angle $\theta_{i}(t)$, the number of undercharged cluster heads $N_{ss}(\theta_{i}(t))$ within the sector, with angle range of $\left(\theta_{i}(t)-B)\sim (\theta_{i}(t)+B\right)$, is considered to determine the movement of the charger. Then, the sum of the power $P_{ss}^{A}(\theta_{i}(t)),$ received by the undercharged cluster heads within the *i*-th sector, is given as

(8) $$\begin{array}{l} P_{ss}^{A}(\theta_{i}(t))=\sum_{s=1}^{N_{SS}(\theta_{i}(t))}P_{r}^{A}({\vec{r}}_{is}(t),{\vec{r}}_{charger}(t)+\Delta {\vec{r}}_{\theta_{i}(t)},\phi_{is}(t))\; \end{array}$$

where $i=1,...,N_{uch}(t)$ and $s=1,...,N_{ss}(\theta_{i}(t))$ and $N_{uch}(t)\geq N_{ss}(\theta_{i}(t))$. The ${\vec{r}}_{is}(t)=(x_{is}(t)\;y_{is}(t))$ is the location of the *s*-th undercharged cluster head within the *i*-th sector and the $\Delta {\vec{r}}_{\theta_{i}(t)}=(r_{m}\cos\theta_{i}(t)\;r_{m}\sin\theta_{i}(t))$ is the incremental vector from the location of the charger to a point on the next location circle at angle $\theta_{i}(t).$ Note that the locations of the undercharged cluster heads are functions of time *t*, since the undercharged cluster heads gradually become overcharged over time.

The next optimal location on the next location circle in terms of optimal $\theta_{opt}(t)$ can be mathematically expressed as

(9) $$\begin{array}{l} \theta_{opt}(t)=\underset{\theta_{i}(t)}{\arg\max}P_{ss}^{A}(\theta_{i}(t))\\ \;=\underset{\theta_{i}(t)}{\arg\max}\sum_{s=1}^{N_{SS}(\theta_{i}(t))}P_{r}^{A}({\vec{r}}_{is}(t),{\vec{r}}_{charger}(t)+\Delta {\vec{r}}_{\theta_{i}(t)},\phi_{is}(t))\\ \;=\;\underset{\theta_{i}(t)}{\arg\max}\sum_{s=1}^{N_{SS}(\theta_{i}(t))}g(\phi_{is}(t))\frac{\alpha }{{\left(d_{is}^{\theta }(t)+\beta \right)}^{2}} \end{array}$$

where $d_{is}^{\theta }(t)=\sqrt{{\left(x_{is}(t)-x_{charger}(t)-r_{m}\cos\theta_{i}(t)\right)}^{2}+{\left(y_{is}(t)-y_{charger}(t)-r_{m}\sin\theta_{i}(t)\right)}^{2}}.$ The $\phi_{is}(t)$ is the orientation angle of the *s*-th undercharged cluster head in the *i*-th sector, adjusted by $\phi_{is}(t)=0^{\circ }$ when $\phi =\theta_{i}(t).$ The $P_{ss}^{A}(\theta_{opt}(t))$ obtained with $\theta_{opt}(t)$ is compared with $P_{ss}^{A}(\theta_{opt}(t-\Delta t)),$ which corresponds to the current location of the charger with optimal $\theta_{i}(t-\Delta t)$ at t−Δt, and the optimal action between staying and shifting is taken. The $\max(P_{ss}^{A}(\theta_{opt}(t)),$ $P_{ss}^{A}(\theta_{opt}(t-\Delta t))$) corresponds to the integrand of Equation (7b). The $\phi_{is}(t)$ in Equation (9) can be obtained from $({\vec{r}}_{i}(t)-{\vec{r}}_{charger}(t)){\left({\vec{r}}_{is}(t)-{\vec{r}}_{charger}(t)\right)}^{T}$ as follows

(10) $$\phi_{is}(t)=\arccos\left[\frac{((x_{i}(t)-x_{charger}(t))\;(y_{i}(t)-y_{charger}(t))){\left((x_{is}(t)-x_{charger}(t))\;(y_{is}(t)-y_{charger}(t))\right)}^{T}\;}{\sqrt{{\left(x_{i}(t)-x_{charger}(t)\right)}^{2}+{\left(y_{i}(t)-y_{charger}(t)\right)}^{2}}\sqrt{{\left(x_{is}(t)-x_{charger}(t)\right)}^{2}+{\left(y_{is}(t)-y_{charger}(t)\right)}^{2}}}\right]$$

In the case of the omnidirectional antenna, Equation (9) can be modified as

(11) $$\begin{array}{l} \theta_{opt}(t)=\underset{\theta }{\arg\max}P_{SC}(t)=\underset{\theta }{\arg\max}\sum_{s=1}^{N_{SC}}P_{r}({\vec{r}}_{s}(t),{\vec{r}}_{charger}(t)+\Delta {\vec{r}}_{\theta })\\ \;=\underset{\theta }{\arg\max}\sum_{s=1}^{N_{SC}}\frac{\alpha }{{\left(d_{s}^{\theta }(t)+\beta \right)}^{2}} \end{array}$$

where $d_{s}^{\theta }(t)=\sqrt{{\left(x_{s}(t)-x_{charger}(t)-r_{m}\cos\theta \right)}^{2}+{\left(y_{s}(t)-y_{charger}(t)-r_{m}\sin\theta \right)}^{2}}.$ In Equation (11), the continuous angle θ is used instead of the discrete angle $\theta_{i}(t)$ and the dependence of the directive gain on angle *φ* no longer exists. To make a decision on the next action, the service circle is considered instead of the service sector. The $P_{sc}(\theta_{opt}(t))$ obtained with $\theta_{opt}(t)$ is compared with $P_{sc}(\theta_{opt}(t-\Delta t))$ and the optimal action between staying and shifting is taken.

Equation (9) implies that the optimal direction of the next movement of the charger at time *t* is the direction toward the undercharged cluster head at the angle $\theta_{opt}(t)$ associated with a sector, where the amount of energy to be provided to the undercharged cluster heads within the sector is the largest among all the sectors associated with all the $\theta_{i}(t)s$, *i* = 1, …, $N_{uch}(t).$ Note here that the evaluation of the amount of energy provided to the sectors is made at the next location circle, so the next movement of the charger is to a point on the next location circle with θ= $\theta_{opt}(t)$. The orientation angle $\phi_{is}(t)$ in Equation (10) represents the orientation angle of the *s*-th undercharged cluster head within the *i*-th sector. The orientation angle $\phi_{is}(t)$ is obtained with reference to the orientation angle $\phi_{is}(t)=0^{\circ }$ when $\phi =\theta_{i}(t).$ With omnidirectional antenna, directive gain according to the orientation angle is uniform. Since the angle range of the sector with omnidirectional antenna is 360°, as described in the paragraph before Equation (8), the sector becomes a circle and thus the $\theta_{opt}(t)$ in Equation (11) is the direction determined by all the undercharged cluster heads, rather than fraction of undercharged cluster heads in a particular sector.

**Figure 5:**
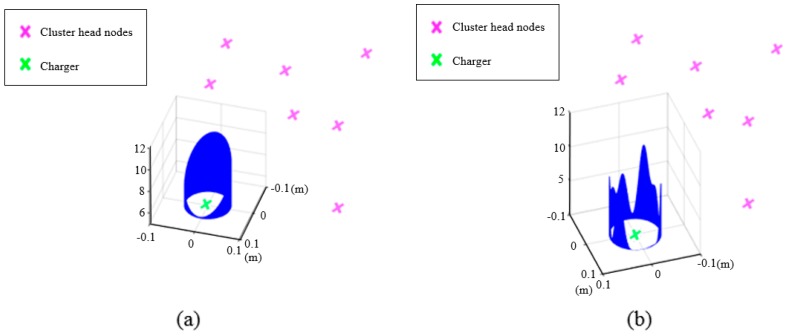
Variation in the power received by undercharged cluster heads according to angle θ: (**a**) $P_{sc}(\theta )$ with an omnidirectional antenna; and (**b**) $P_{ss}^{A}(\theta )$ with a directional antenna of 12 dB antenna gain.

As predicted by Figure 4b, the smaller beamwidth affects fewer member nodes during charging by the mobile charger, which in turn increases the time taken for energy trading in the second stage. Therefore, it is necessary to increase the target energy level $EL_{\min}^{A}$ with higher antenna gain. In order to figure out the typical variation in $P_{sc}(\theta )$ along θ with an omnidirectional antenna, and the variation in $P_{ss}^{A}(\theta )$ with a directional antenna of 12 dB antenna gain, potential variations in the next location circle are illustrated, as shown in Figure 5. The $P_{sc}(\theta )$ along θ in Figure 5a shows uni-modality while the $P_{ss}^{A}(\theta )$ results in multi-modality. This is because with the omnidirectional antenna the geometrical distance of the closest undercharged cluster head basically determines the $P_{sc}(\theta )$, whereas, with directional antennas, the orientation angles as well as the geometrical distances of the undercharged cluster heads determine the $P_{ss}^{A}(\theta )$. Because those two variations of received power produce different characteristics, two algorithms are used to determine the optimal θ. For the omnidirectional antenna, the GSS [29] is used, which is appropriate for finding an optimal solution with a uni-modal function, whereas, for directional antennas, a sector-based search based on Equation (9) is used.

**Figure 6:**
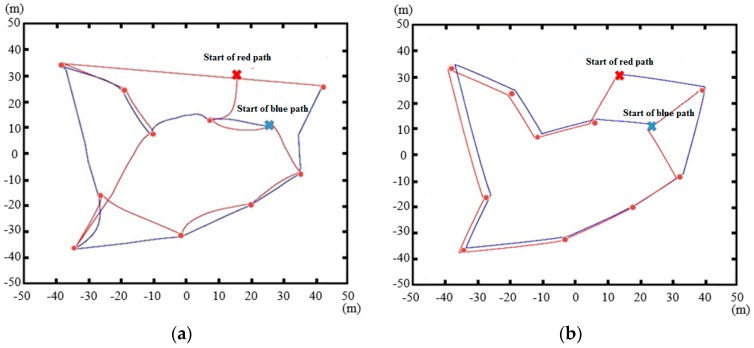
Two different charging paths with different starting points: (**a**) omnidirectional antenna; and (**b**) directional antenna with 12 dB antenna gain.

**Figure 7:**
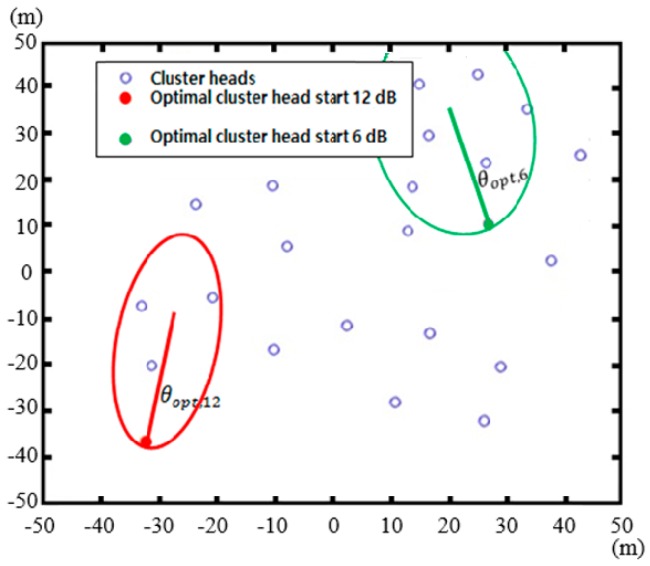
Optimal starting points with different antenna gains. Optimal starting points for directional antennas are often located on the outskirts of the total service area.

Figure 6 presents the paths of the mobile charger with an omnidirectional antenna, and a directional antenna with 12 dB antenna gain, when the charger starts from two different locations with each antenna. The small red circles represent cluster heads. As shown in Figure 6a, the paths created with the omnidirectional antenna consist of smooth curves, in contrast to the line segments associated with the directional antenna. With the omnidirectional antenna, the configuration of undercharged cluster heads within the service circle can affect the direction of the next movement of the mobile charger. With uniform directive gain of omnidirectional antenna over the entire range of the orientation angle, two or more undercharged cluster heads can affect the direction of the next movement. This can cause a transition of the charger along a smooth curve. In contrast, the direction of the movement of the charger with directional antenna hardly changes, once the orientation angle 0° of the directional antenna, e.g., the orientation angle providing the highest directive gain, is set to the direction of a particular undercharged cluster head. As a result, $T_{cm}^{A}(PATH_{i})$ in Equation (7a) for the directional antenna of 12 dB antenna gain is significantly smaller. Both figures show that the mobile charger moves from one cluster head to another, since this is the most efficient way to charge the cluster heads over time. The paths of the directional antenna with two starting positions are similar, while the two paths for the omnidirectional antenna show a substantial disparity. This indicates that the starting point is significantly more important for the omnidirectional antenna. In the case of the directional antenna, the charger is more likely to be guided to the closest cluster head along a straight line segment regardless of the starting point. It is worth mentioning that the optimal starting points of the mobile charger are typically located on the outskirts of the total service area, as shown in Figure 7. This is because more sensor nodes fall into the sector than in the case of a centrally located charger. However, with the omnidirectional antenna this trend becomes weak. Figure 6 shows sample starting points with directional antennas. In the simulations, all the cluster head locations were taken as starting points, and their respective total charging times were evaluated to determine the smallest one.

### 2.6. Energy Trading in the Second Stage

**Figure 8:**
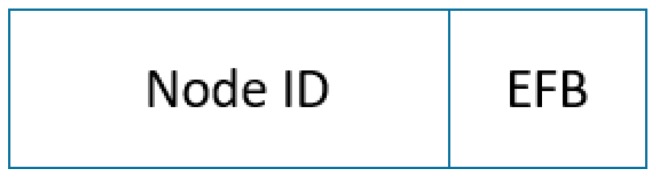
Message broadcasted by the buyer nodes stating a need for energy.

Energy trading begins when all of the cluster heads become overcharged. During the first charging stage, many member nodes also become overcharged. However, some member nodes are still undercharged. To bring these undercharged member nodes to the target level *ET_s_*, energy trading is conducted, in which all the overcharged sensor nodes, including the cluster heads, act as seller nodes and all the undercharged sensor nodes solicit energy as buyer nodes. This process achieves energy balancing for all of the sensor nodes. All the sensor nodes are assumed to employ omnidirectional antennas and are bi-directional in transacting energy. When the mobile charger finishes charging, it broadcasts a message indicating termination of charging. Then, all the undercharged member sensor nodes, knowing their SoC, send broadcast messages by using frequencies other than the charging frequency in a time slotted fashion similar to the one by Usman et al. [30], as shown in Figure 8.

The Node ID indicates the ID number of the member node, and the EFB is the energy flag bit. If the EFB is set to 1, the node is still undercharged. The buyer nodes keep receiving energy from the seller nodes and can become overcharged. When the buyer nodes become overcharged, they broadcast the message, as in Figure 8, with the EFB set to 0. Every overcharged sensor node acts as a seller node as long as it has surplus energy. As the seller node transmits surplus energy only, it cannot become a buyer node again.

During energy trading, many-to-one correspondences are established. The amount of energy increment of the *b*-th buyer node during the small time interval $\Delta t_{b}$ is given by

(12) $$E_{b}(t+\Delta t_{b})=E_{b}(t)+\sum_{i\in S}P_{r}({\vec{r}}_{b},{\vec{r}}_{s_{i}})\Delta t_{b}\;$$

where $P_{r}({\vec{r}}_{b},{\vec{r}}_{s_{i}})$ expressed by Equation (2a) is the power received from the *i*-th seller node, and *S* indicates the set of seller nodes over the time interval $\Delta t_{b}$ from time *t*. Since all the seller nodes transmit energy omnidirectionally, they also receive energy from other seller nodes during energy trading. All the losses and gains of the *i*-th seller node during the small time interval $\Delta t_{s}$ are given by

(13) $$\begin{array}{l} E_{s_{i}}(t+\Delta t_{s})=E_{s_{i}}(t)-\frac{P_{r0}\Delta t_{s}}{T_{f}}+\sum_{j\in S^{\prime }}P_{r}({\vec{r}}_{s_{i}},{\vec{r}}_{s_{j}})\Delta t_{s}\\ =E_{s_{i}}(t)-\frac{\left(\alpha /\beta^{2}\right)}{T_{f}}\Delta t_{s}\cdot u(E_{s_{i}}(t)-ET_{s})+\sum_{j\in S^{\prime }}\frac{\alpha }{{\left(d_{ij}+\beta \right)}^{2}}\Delta t_{s}\; \end{array}$$

where *s_j_* represents the *j*-th seller node and the parameter $T_{f}=(\alpha /\beta^{2})/P_{t}$, the ratio of received power with distance *d* = 0 m to transmit power *P_t_* of the seller node, indicates charging efficiency. The $P_{r0}=(\alpha /\beta^{2})$ is obtained from Equation (2a) with *d_j_*(*t*) = 0 m. The function *u*(·) is the unit step function. As the transmit power of the charger is implicit in the power Equation (2a), the charging efficiency *T_f_* for RF band is adopted from [31,32]. The $S^{\prime }$ indicates the set of seller nodes, excluding the *i*-th seller node, over the time interval $\Delta t_{s}$ from time *t*, and the *d_ij_* represents the distance between the *i*-th and *j*-th seller nodes. The new seller node loses and gains energy according to Equation (13). The sets *S* and S′ are updated whenever a new seller node is added. The end of energy trading between sensor nodes can be detected by them, based on the comparison between the number of broadcast messages with EFB = 0 and the number of broadcast messages with EFB = 1. If they are equal, it indicates the end of energy trading. In the event of packet errors or loss while broadcasting messages, the energy trading can be affected. Since seller nodes keep transmitting energy until the end of energy trading, the escape condition on energy trading must be satisfied. If the number of broadcast messages with EFB = 0 is not equal to that with EFB = 1, because of the event of packet errors, the energy trading might be continued until either: (1) all the sensor nodes reach the energy level *ET_S_* when the energy threshold for cluster heads is appropriately set; or (2) some member sensor nodes cannot reach the energy level *ET_S_* when the energy threshold for cluster heads is not appropriately set, e.g., $EL_{\min}^{A}$ < *ET_S_*.

## 3. Simulation Results

The size of the total service area is 100 m × 100 m, and the number of different distributions of sensor nodes for each combination of the antenna gain and the number of sensor nodes *N_T_* is 100. The clustering process and path planning are all performed by the charger, as aforementioned. All the sensor nodes are initially energy-depleted. For each antenna gain, the total charging time and the time taken for energy trading are obtained over the range of *N_T_* = 100~300. While the target energy level of the cluster heads $EL_{\min}^{A}$ is adjusted according to the type of directional antenna and charging efficiency *T_f_*, every sensor node must be charged above the target energy level *ET_s_* = 2 Joules. Other simulation parameters, such as the total service area and the parameters of power equation, Equation (2a), are set to values identical to those in [10]. Parameters α and β of the power equation are set to 36 and 30, respectively. As the power equation is identical to that in [10], the difference in the total amount of energy provided to the sensor network is linearly proportional to (total charging time by the scheme in [10]–[total charging time by hybrid charging scheme–time taken for energy trading by hybrid charging scheme]). Note that the energy trading simply implies the re-distribution of energy provided to the sensor network. For the charging efficiency *T_f_*, two values are used. By considering the experimental results in [31] with an RF band 1~2 GHz, *T_f_* = 0.05 is used. In addition, for comparison, *T_f_* = 0.01 is used. Table 1 presents the simulation parameters. All the threshold values for the charging by the mobile charger are chosen to ensure that the sensor nodes have enough energy for successful energy trading. The $EL_{\min}^{A}$ is set higher with higher antenna gain, since a smaller number of overcharged sensor nodes is obtained while charging the cluster heads. Furthermore, the required $EL_{\min}^{A}$ with high antenna gain 12 dB is higher with a smaller number of sensor nodes because of a smaller number of overcharged sensor nodes. Other simulation parameters are as follows. The radii of the inclusion circle and service sector are set to *R_cl_* = 10 m and *R_s_* = 40 m, respectively. The tolerance parameter for the GSS is set to 10^−4^, which corresponds to a 3° angle spacing.

**Figure 9:**
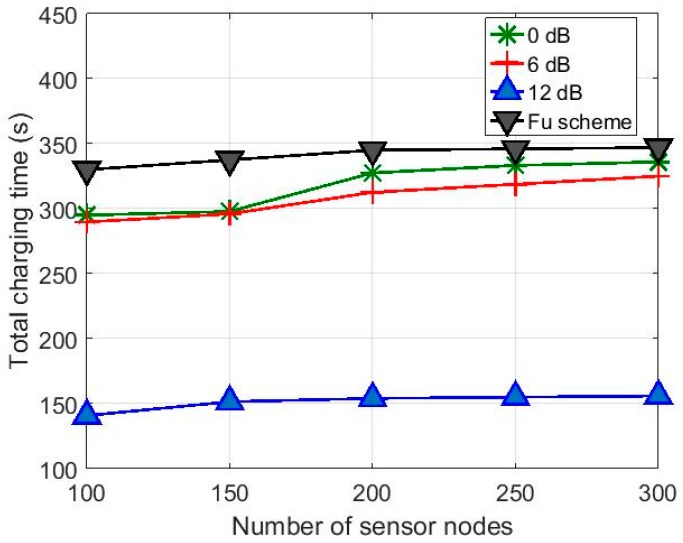
Variation of total charging time according to number of sensor nodes with different antenna gains for *T_f_* = 0.05.

**Figure 10:**
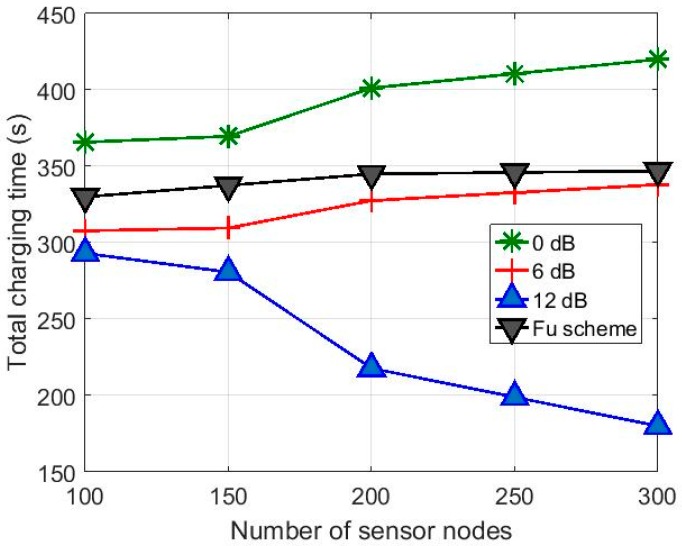
Variation of total charging time according to number of sensor nodes with different antenna gains for *T_f_* = 0.01.

Figure 9 shows the total charging time *T_tot_* for the hybrid charging scheme with the charging efficiency *T_f_* = 0.05 compared with the results in [10]. It is seen in Figure 9 that the hybrid charging scheme is more advantageous than that in [10] in terms of charging time. Considering that the *T_tot_* of the hybrid charging scheme includes the time taken for energy trading, the total amount of energy provided by the mobile charger to the sensor network is significantly smaller to achieve the same *ET_s_* for all the sensor nodes. Figure 10 shows the simulation results with *T_f_* = 0.01. Even with the lower charging efficiency, the hybrid charging scheme with the directional antennas still shows reduced total charging time. The variation in total charging time according to antenna gain is smaller for small *N_T_* values as compared to that in Figure 9. This is because, for the higher $EL_{\min}^{A}$ set for *T_f_* = 0.01, the time required for charging by the charger is increased.

**Figure 11:**
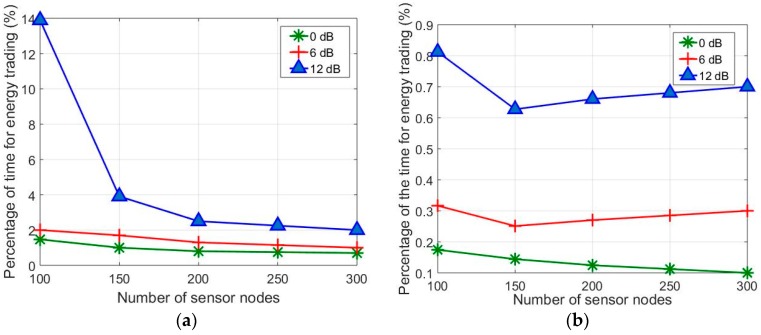
Percentage of time for energy trading over total charging time with different antenna gains and charging efficiency: (**a**) *T_f_* = 0.05; and (**b**) *T_f_* = 0.01.

Figure 11 presents the proportion of total charging time used for energy trading. With *T_f_ =* 0.05, the proportion of time for energy trading is increased with the higher antenna gain, as expected. That is, with 12 dB antenna gain, the proportion is approximately 10% higher than that with 0 dB antenna gain for *N_T_* = 100, and it decreases with increasing *N_T_*. In fact, higher density of sensor nodes increases the amount of captured RF energy, thus decreasing the time for energy trading. The significant reduction observed for the 12dB antenna gain is not observed with the other antenna gains. With *T_f_ =* 0.01, the proportion of time for energy trading is much smaller because there are substantially larger number of overcharged seller nodes. The $EL_{\min}^{A}$ values, which are set considerably higher for *T_f_ =* 0.01, cause a significantly larger number of overcharged sensor nodes. The higher $EL_{\min}^{A}$ value set for lower *T_f_* overpowers the compensating effect of the lower *T_f_*, leading to a smaller proportion of time for energy trading. As seen in Figure 11a,b, a larger number of sensor nodes are, in general, beneficial for reducing the proportion of time required for energy trading.

**Figure 12:**
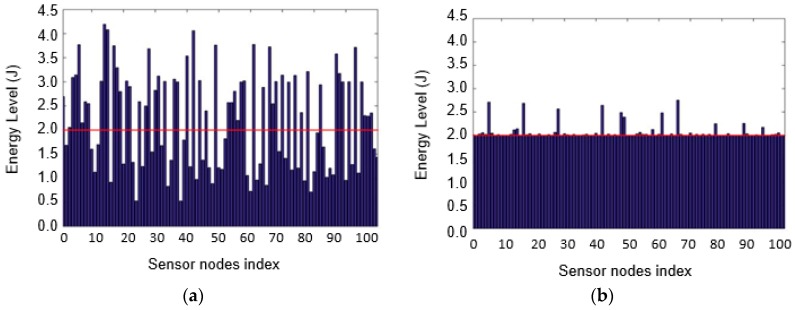
Energy level profile over sensor nodes with 12 dB antenna gain and *T_f_* = 0.05: (**a**) before energy trading; and (**b**) after energy trading.

**Figure 13:**
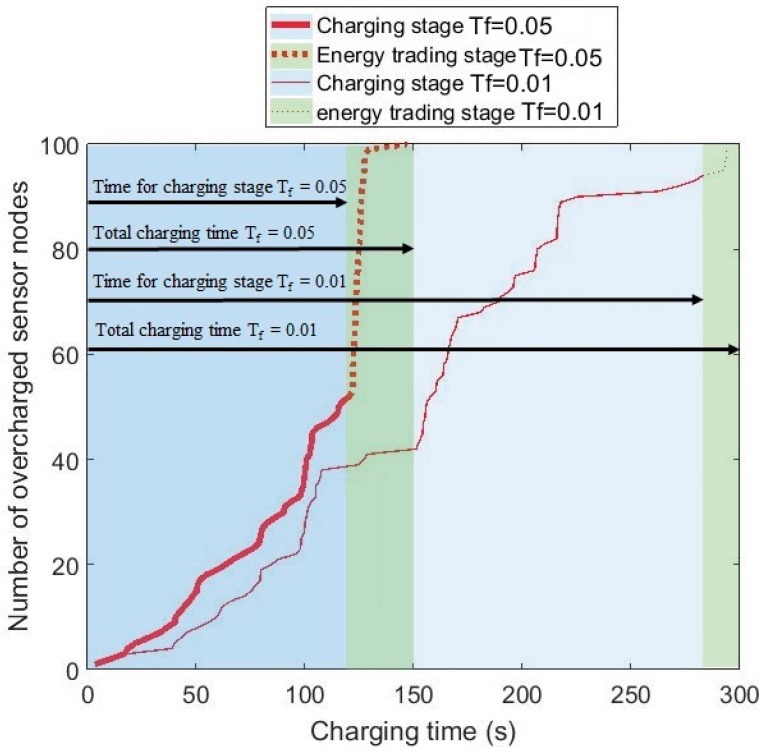
Number of overcharged sensor nodes as a function of time with 12 dB antenna gain for *T_f_* = 0.01 and *T_f_* = 0.05.

The energy profiles of the sensor nodes before and after the energy trading can be seen in Figure 12 with 12 dB antenna gain and the charging efficiency *T_f_* = 0.05. As seen in Figure 12b, no sensor nodes remain undercharged after energy trading. With the simulation parameters shown in Table 1, all the sensor nodes are charged over the target energy level *ET_s_* = 2 Joules. Figure 12a shows that the energy levels of the overcharged sensor nodes, including cluster heads, are significantly higher than those of the undercharged sensor nodes. The behavior of the hybrid charging scheme is shown in Figure 13 in an integrated fashion. Figure 13 shows the variation of the number of overcharged sensor nodes over time with 12 dB antenna gain and *N_T_* = 100. The two plots in the figure support the previous discussion regarding the time needed for charging by the mobile charger and the time for energy trading. The number of overcharged sensor nodes created in the first stage with T_f_ = 0.01 is considerably larger due to higher $EL_{\min}^{A}$. This results in a reduced time for energy trading, which is also observed in Figure 11.

## 4. Conclusions

The main advantage of the proposed hybrid charging scheme is the division of the entire charging process into two stages: charging by the mobile charger and energy trading for balancing. The mobile charger is only concerned with the cluster heads, and thus moves over a shorter path, consequently spending less time for charging. During energy trading, since there are more seller nodes than buyer nodes, energy transfer is performed through many-to-one correspondences. The many-to-one correspondences enable all the sensor nodes in the wireless sensor network to be charged over the target level in a relatively short time. The impact of higher antenna gain is beneficial for reducing the time needed to charge the cluster heads, but subsequently increases the proportion of time needed for energy trading. Nevertheless, the proportion of time needed for energy trading is considerably smaller than the time required for charging by the mobile charger, providing a significant reduction with higher antenna gain in total charging time as well as total charging energy.

## Figures and Tables

**Table 1:** Simulation parameters.

Charging Efficiency		$EL_{\min}^{A}$ for 0 dB		$EL_{\min}^{A}$ for 6 dB		$EL_{\min}^{A}$ for 12 dB	
	Thresholds						
*T_f_* = 0.01		3.5 J	300 nodes	3.5 J	300 nodes	4.5 J	300 nodes
		3 J	250 nodes	3 J	250 nodes	4.5 J	250 nodes
		2.5 J	200 nodes	3 J	200 nodes	6 J	200 nodes
		2.5 J	150 nodes	3 J	150 nodes	7.5 J	150 nodes
		2.5 J	100 nodes	3 J	100 nodes	7.5 J	100 nodes
*T_f_* = 0.05		2 J	300 nodes	2.5 J	300 nodes	3 J	300 nodes
		2 J	250 nodes	2.5 J	250 nodes	3 J	250 nodes
		2 J	200 nodes	2.5 J	200 nodes	3 J	200 nodes
		2 J	150 nodes	2.5 J	150 nodes	3 J	150 nodes
		2 J	100 nodes	2.5 J	100 nodes	3 J	100 nodes
